# *TARDBP* mutations in a cohort of Italian patients with Parkinson’s disease and atypical parkinsonisms

**DOI:** 10.3389/fnagi.2022.1020948

**Published:** 2022-09-29

**Authors:** Cinzia Tiloca, Stefano Goldwurm, Narghes Calcagno, Federico Verde, Silvia Peverelli, Daniela Calini, Anna Lena Zecchinelli, Davide Sangalli, Antonia Ratti, Gianni Pezzoli, Vincenzo Silani, Nicola Ticozzi

**Affiliations:** ^1^Department of Neurology and Laboratory of Neuroscience, IRCCS Istituto Auxologico Italiano, Milan, Italy; ^2^Parkinson Institute of Milan, ASST Gaetano Pini/CTO, Milan, Italy; ^3^Neurology Residency Program, Università degli Studi di Milano, Milan, Italy; ^4^Department of Pathophysiology and Transplantation, “Dino Ferrari” Center, Università degli Studi di Milano, Milan, Italy; ^5^Department of Neurology – Stroke Unit, A. Manzoni Hospital – ASST Lecco, Lecco, Italy; ^6^Department of Medical Biotechnology and Translational Medicine, Università degli Studi di Milano, Milan, Italy

**Keywords:** *TARDBP*, TDP-43, Parkinson’s disease, atypical parkinsonism, genetics

## Abstract

**Background:**

Aggregates of TAR DNA-binding protein of 43 kDa (TDP-43) represent the pathological hallmark of most amyotrophic lateral sclerosis (ALS) and of nearly 50% of frontotemporal dementia (FTD) cases but were also observed to occur as secondary neuropathology in the nervous tissue of patients with different neurodegenerative diseases, including Parkinson’s disease (PD) and atypical parkinsonism. Mutations of *TARDBP* gene, mainly in exon 6 hotspot, have been reported to be causative of some forms of ALS and FTD, with clinical signs of parkinsonism observed in few mutation carriers.

**Methods:**

Direct DNA sequencing of *TARDBP* exon 6 was performed in a large Italian cohort of 735 patients affected by PD (354 familial and 381 sporadic) and 142 affected by atypical parkinsonism, including 39 corticobasal syndrome (CBS) and 103 progressive sopranuclear palsy (PSP). Sequencing data from 1710 healthy, ethnically matched controls were already available.

**Results:**

Four *TARDBP* missense variants (p.N267S, p. G294A, p.G295S, p.S393L) were identified in four patients with typical PD and in two individuals with atypical parkinsonism (1 CBS and 1 PSP). None of the detected mutations were found in healthy controls and only the variant p.N267S was previously described in association to idiopathic familial and sporadic PD and to CBS.

**Conclusion:**

In this study we provide further insight into the clinical phenotypic heterogeneity associated with *TARDBP* mutations, which expands beyond the classical ALS and FTD diseases to include also PD and atypical parkinsonism, although with a low mutational frequency, varying considerably in different Caucasian populations. In addition, our study extends the spectrum of *TARDBP* pathogenetic mutations found in familial and sporadic PD.

## Introduction

TAR DNA-binding protein of 43 kDa (TDP-43), encoded by *TARDBP* gene, was originally described as the major component of ubiquitin-positive neuronal inclusions in nearly all (97%) amyotrophic lateral sclerosis (ALS) cases and in nearly 50% of patients with frontotemporal degeneration (FTLD-TDP), while most of the remaining cases have tau pathology (FTLD-tau). As such, ALS and frontotemporal dementia (FTD) are now recognized as opposite ends of the same disease continuum (Ling et al., 2013). Mutations of *TARDBP* gene were initially identified as causative of ALS, with a prevalence of 5% in familial and 1–2% in sporadic ALS cases (Sreedharan et al., 2008; Lattante et al., 2013), but ranging widely across populations from 0 to 12 and 0 to 5%, respectively (Gitcho et al., 2008; Guerreiro et al., 2008; Kabashi et al., 2008; Corrado et al., 2009; Gijselinck et al., 2009; Piaceri et al., 2012; Bertolin et al., 2014; Narain et al., 2018). Other studies detected *TARDBP* mutations also in FTD and ALS-FTD cases, although with a very low frequency (Benajiba et al., 2009; Borroni et al., 2009, 2010; Praline et al., 2012). Mutations in *TARDBP* gene predominantly map in exon 6 which encodes for the C-terminal region, a glycin-rich low complexity domain with a crucial role in protein-protein interaction, nucleo-cytoplasmic shuttling and aggregation propensity (Ticozzi et al., 2012). Interestingly, autoptic studies of ALS and FTD cases have shown a widespread distribution of TDP-43 aggregates through the whole central nervous system (CNS), suggesting a four-stage model of spreading of TDP-43 pathology with disease progression (Brettschneider et al., 2013). In stage 1, TDP-43 inclusions mainly occur in the projection neurons of the agranular motor cortex and lower motor neurons of brainstem and spinal cord, while in stage 2 they are observed in the reticular formation and parvocellular portion of the red nucleus. In stages 3 and 4, TDP-43 aggregates involve the pre-frontal areas, the striatum and the allocortical regions, providing a biological basis for the development of additional clinical manifestations observed in these patients. Indeed, although poorly reported in the past years, extrapyramidal features such as backward falls, retropulsion, bradykinesia, rigidity, and postural instability are now increasingly observed in ALS cases (Pasquini et al., 2022), being relevant for therapeutic treatment, rehabilitation and physiotherapy strategies. Nigrostriatal system dysfunction has been observed also by neuroimaging studies not only in ALS with parkinsonism (ALS-P) but also in ALS cases without clinical signs of parkinsonism, with substantia nigra hyperechogenicity reported by transcranial sonography in 67% of patients with sporadic ALS (Takahashi et al., 1993; Fathinia et al., 2013). In patients affected by the behavioral variant of FTD (bvFTD), parkinsonism represents a frequent clinical presentation, with symptoms like bradykinesia, parkinsonian gait, rigidity, and abnormal posture occurring before, during or after the onset of behavioral and cognitive changes (Baizabal-Carvallo and Jankovic, 2016). Significantly, the possibility of a common pathogenic mechanism affecting ALS/FTD and other neurodegenerative diseases has been suggested also by the finding of TDP-43 positive aggregates in the nervous tissue of patients suffering from alpha-synucleinopathies [Parkinson’s Disease (PD), dementia with Lewy Bodies (DLB), and multiple system atrophy (MSA)], tauopathies [progressive sopranuclear palsy (PSP) and corticobasal syndrome (CBS)] Alzheimer’s disease and Huntington’s disease (Chen-Plotkin et al., 2010).

Consistent with evidence indicating a broad role of TDP-43 in neuronal degeneration and with the co-existence of parkinsonism and ALS/FTD in few *TARDBP* mutation carriers, previous studies were conducted to investigate the role of *TARDBP* mutations in PD and other parkinsonisms (PSP and CBS). Three independent studies screened PD cohorts of Caucasian American (*n* = 463), French Canadian (*n* = 125), and Dutch (*n* = 429) origin, but failed to detect mutations (Kabashi et al., 2009; Ticozzi et al., 2011; van Blitterswijk et al., 2013). Nevertheless, the first direct link between *TARDBP* and PD was found in a cohort of PD patients of Sardinian descent, where the p.A382T variant was observed in 8 cases of sporadic PD cases and a family with atypical parkinsonism, with a reported mutational frequency of 2.5% (Quadri et al., 2011). This mutation is the most frequent *TARDBP* variant among Italian ALS cases and is especially common in Sardinia due to a founder effect, where it accounts for more than one third of all ALS cases (Chiò et al., 2011; Orrù et al., 2012; Borghero et al., 2014). Interestingly, parkinsonism has been reported as a clinical manifestation in some of these cases. Another report identified a single sporadic PD case in Southern Italy carrying the p.N267S mutation (Gagliardi et al., 2018), while a screening of a North American cohort found the same mutation in one familial PD case (Rayaprolu et al., 2013). Most ALS cases with *TARDBP* mutations manifesting parkinsonism were reported in the Italian population (Borghero et al., 2011; Mosca et al., 2012; Ticozzi et al., 2013; Pasquini et al., 2022) except for one Japanese pedigree. More recently, the first *TARDBP* mutation outside exon 6 manifesting with parkinsonism was identified in a Chinese ALS family (Chen et al., 2021).

Here, we describe the results of a mutational screening of *TARDBP* exon 6 in a large Italian cohort of patients with a clinical diagnosis of PD (*n* = 735) and atypical parkinsonism (*n* = 142).

## Materials and methods

### Patients

The study cohort consisted of 735 patients with PD (443 males and 292 females, mean age at onset 57.8 ± 10.2 years) and 142 with atypical parkinsonism (77 males and 65 females, mean age at onset 65.1 ± 7.1 years). The latter group included 39 CBS and 103 PSP cases. All patients were individuals of Italian origin, who were consecutively recruited at Parkinson Institute, ASST Gaetano Pini-CTO and contributed to the Parkinson Institute Biobank.^1^ Clinical diagnosis was made by neurologists experienced in the field of movement disorders according to internationally recognized criteria (Postuma et al., 2015; Höglinger et al., 2017; Jabbari et al., 2020). For each patient the following information were collected: gender, age of onset, disease duration at last examination, clinical features. Within the study cohort, 354 PD patients and 12 individuals with atypical parkinsonism presented a positive family history (Supplementary Table 1), defined as at least one first- or second-degree relative having PD.

### Healthy controls

Publicly available genetic data from 1710 Italian individuals (non-Sardinians) without known neurological disorders previously screened for TARDBP exon 6 mutations were used as control data (Corrado et al., 2009; del Bo et al., 2009; Origone et al., 2010; Conforti et al., 2011; Gagliardi et al., 2018).

### Genetic analysis

Genomic DNA was isolated from peripheral blood according to standard procedures. The *TARDBP* exon 6 and the exon-intron boundary regions were amplified by polymerase chain reaction (PCR) as previously described (Corrado et al., 2009). PCR products were purified enzimatically by using illustra™ ExoStar™ (GE Healthcare, Boston, MA, United States) and analyzed by direct sequencing using BigDye Terminator v3.1 Cycle Sequencing Kit (Thermo Fisher Scientific, Waltham, MA, United States) on 3500 Genetic Analyzer (Applied Biosystems, Waltham, MA, United States). All identified variants were confirmed by sequencing an independent PCR product. The following splicing analysis tools were used to assess the possible effects of identified variants on splicing regulatory sites: Human Splicing Finder, NNSplice, MaxEntScan, GeneSplicer, Splice site analysis-SSF, ESE Finder. Nucleotide numbering of *TARDBP* gene variants reflects cDNA numbering with + 1 corresponding to the A of the ATG translation initiation codon in reference sequence NM_007375.3. PD patients enrolled in this study were previously screened for major PD-related genes, including *LRRK2*, *Parkin*, *PINK1*, *DJ1*, and 45 were found to carry mutations in these genes.

### Ethical standards and data availability

Written informed consent for genetic studies was obtained from individuals recruited by the “Parkinson Institute Biobank,” member of the Telethon Network of Genetic Biobank. This study was approved by the ethical committee of IRCCS Istituto Auxologico Italiano (project DAMARE) and was performed in accordance with the 1964 Declaration of Helsinki and its later amendments. Pseudo-anonymized datasets analyzed for this study are archived on Zenodo (10.5281/zenodo.6985371) and will be shared upon reasonable request.

## Results

Genetic analysis of the mutational hotspot exon 6 of the *TARDBP* gene was performed in 735 patients with PD (354 familial and 381 sporadic) and 142 patients with atypical parkinsonism (39 CBS and 103 PSP). We identified six cases carrying *TARDBP* missense variants, all occurring in heterozygous state (Table 1 and Supplementary Figure 1).

**TABLE 1:** *TARDBP* missense variants identified in our patient cohort.

Mutation*	Mutation carriers (present study)	MAF		ACMG classification	Reported associated phenotypes (N. patients^§^)	Functional evidences	References
		Italian controls^†^ (*n* = 1,710)	gnomAD^‡^ (*n* = 104,052)				
p.N267S c.800A > G	PD (*n* = 1) PSP (*n* = 1) CBS (*n* = 1)	0	8.2 × 10^–5^	Likely pathogenic	ALS (*n* = 3) FTD (*n* = 2) PD (*n* = 2) CBS (*n* = 1) LMND (*n* = 1)	Decreased protein expression in patient’s lymphoblastoid cells	Corrado et al., 2009; Borroni et al., 2010; Rayaprolu et al., 2013; Gagliardi et al., 2018; Narain et al., 2018
p.G294A c.881G > C	PD (*n* = 1)	0	0	Pathogenic	ALS (*n* = 1)	Increased intracellular aggregates Differences in stress granules formation during sorbitol-induced stress	Sreedharan et al., 2008; Nonaka et al., 2009; Dewey et al., 2011
p.G295S c.883G > A	PD (*n* = 1)	0	0	Pathogenic	ALS (*n* = 16) FTD/ALS (*n* = 2)	Increased neurotoxicity Ability to disrupt liposome integrity	(Benajiba et al., 2009; Corrado et al., 2009; Piaceri et al., 2012; Bertolin et al., 2014; Borghero et al., 2014; Sun et al., 2014
p.S393L c.1178C > T	PD (*n* = 1)	0	1.1 × 10^–5^	Likely pathogenic	ALS (*n* = 2) PA (*n* = 1)	Age-related increased cell death Age-related abnormalities in mitochondria and lysosomes morphology and motility Aberrant lower molecular weight TDP-43 bands in lymphocytes lysates	Corrado et al., 2009; Origone et al., 2010; Praline et al., 2012; Kreiter et al., 2018

The table summarizes mutational frequencies in the general population, clinical phenotypes previously associated to TARDBP variants and functional data supporting their role in neurodegeneration.

*Numbering of TARDBP variants according to the NCBI Reference Sequence NM_007375.3.

^†^Italian healthy controls screened in five previous TARDBP genetic studies (Corrado et al., 2009; del Bo et al., 2009; Origone et al., 2010; Conforti et al., 2011; Gagliardi et al., 2018).

^‡^MAF was reported according to gnomAD v2.1.1 non-neuro dataset, available at http://gnomad.broadinstitute.org.

^§^Only probands were considered in case of mutated families. ALS, amyotrophic lateral sclerosis; CBS, corticobasal syndrome; FTD, frontotemporal dementia; gnomAD, Genome Aggregation Database; LMND, lower motor neuron disease; MAF, minor allele frequency; PA, progressive anarthria; PD, Parkinson’s disease.

In particular, four different missense mutations (c.800A > G, p.N267S; c.881G > C, p. G294A; c.883G > A, p.G295S; c.1178C > T, p.S393L) were observed in four individuals affected by typical PD (Table 1). Additionally, the mutation p.N267S was also detected in two patients with atypical parkinsonism (1 PSP and 1 CBS) (Table 1). Overall, we observed a mutational frequency for *TARDBP* of 0.7% (6/877) in our cohort of Italian parkinsonian patients. These variants were instead absent in 1710 Italian healthy controls, appear to be extremely rare in public sequencing databases and are classified as pathogenic or likely pathogenic according to the ACMG Criteria (Table 1).

Our mutational analysis also detected three other single nucleotide variants in three PD cases: an intronic variant (c.715-31C > T) and two synonymous variants, respectively p.Gly335Gly (c.1005T > A) and p.Ala341Ala (c.1023C > T), The intronic variant upstream exon 6 (c.715-31C > T) was not expected to induce significant splicing motif alterations by different bioinformatics predictive algorithms (Supplementary Table 2). According to the ESEfinder Tool, no impact on exonic splicing enhancers (ESEs) was predicted for the variant c.1005T > A, while the exonic variant c.1023C > T was predicted to disrupt the putative binding site for the SR protein SRSF1, although its biological effect on TDP-43 splicing activity remains unclear. All these variants are classified as likely benign according to the ACMG Criteria (Supplementary Table 2).

The clinical characteristics of patients carrying *TARDBP* missense mutations are reported in Table 2. Only patient #1149, harboring the p.N267S variant, reported a positive family history, having a brother affected by MSA but unfortunately DNA was not available for segregation analysis. The remaining mutation carriers were all sporadic patients. None of the *TARDBP* mutated patients carried additional mutations in other PD-associated genes.

**TABLE 2:** Demographic and clinical characteristics of patients harboring *TARDBP* mutation in the study cohort.

ID	Mutation	Sex	Diagnosis	Age at onset	Disease duration* (yrs)	Family history	Parkinsonian features				Response to levodopa	DaT-SCAN	Brain MRI	Cognitive deficit	Other clinical features
							Resting tremor	Brady kinesia	Postural instability	Rigidity					
571	p.N267S	F	CBS	74	8	No	No	Yes	Yes	Yes	n/a	n/a	Asymmetrical frontoparietal atrophy (right > left)	Yes	
1149	p.N267S	M	PD	60	13	MSA-P	Yes	Yes	Yes	Yes	Yes	Positive	n/a	Attention, short-term memory, visuospatial functions (12 years after disease onset)	Motor fluctuations and dyskinesia (10 years after disease onset) visual hallucinations (12 years after disease onset)
1196	p.N267S	F	PSP	67	11	No	No	Yes	Yes	Yes	No	n/a	n/a	Yes	
2571	p.S393L	M	PD	67	15	No	No	Yes	No	Yes	Yes	n/a	Aspecific WMLs	No	Motor fluctuations and dyskinesia (8 years after disease onset) impulse control disorder
2709	p.G295S	F	PD	47	7	No	No	Yes	No	Yes	Yes	Positive	n/a	No	
4558	p.G294A	F	PD	58	11	No	Yes	Yes	Yes	Yes	Yes	n/a	n/a	No	Motor fluctuations (10 years)

*From symptom onset to the last follow-up evaluation. CBS, corticobasal syndrome; MSA-P, multiple system atrophy-parkinsonian type; n/a, not available; PD, Parkinson’s disease; PSP, progressive supranuclear palsy; WMLs, white matter lesions.

## Discussion

Parkinsonisms are a group of clinically and pathologically heterogeneous neurodegenerative movement disorders, all characterized by the loss of dopaminergic nigrostriatal neurons and by the presence of typical motor symptoms of tremor, rigidity, bradykinesia, and postural instability. Some reports described a secondary TDP-43 pathology in parkinsonisms (7% of PD, 15.4% of CBS, 6% of PSP), although with a specific distinctive pattern of morphology, regional distribution and severity of TDP-43 aggregates compared to ALS/FTD TDP-43 proteinopathies.

In this study we sequenced exon 6 of *TARDBP* gene in a large cohort of Italian patients affected by different forms of parkinsonism and identified 4/735 (0.5%) PD and 2/142 (1.4%) atypical parkinsonism cases harboring pathogenic mutations. To date, 3,647 PD patients of Caucasian origin have been screened for *TARDBP* mutations by eight distinct genetic studies, including the present report (Kabashi et al., 2009; Quadri et al., 2011; Ticozzi et al., 2011; Cannas et al., 2013; Rayaprolu et al., 2013; van Blitterswijk et al., 2013; Gagliardi et al., 2018). With the exception of the founder p.A382T mutation in the Sardinian population, *TARDBP* variants have been reported to occur at very low rate (from 0 to 0.5%) in PD, with a heterogeneous frequency in different populations. *TARDBP* mutations have been associated with both familial and sporadic PD (literature data reviewed in Supplementary Table 3).

Regarding the *TARDBP* variants identified in our study, only the p.N267S mutation has been previously found in association with idiopathic familial and sporadic PD and to CBS (Huey et al., 2012; Rayaprolu et al., 2013; Gagliardi et al., 2018; Table 1 and Supplementary Table 3). The other three variants, p.G294A, p.G295S, and p.S393L, have been reported as causative mutations only in ALS or FTD cases with or without parkinsonism, but they have never been described in association with PD and PD-like phenotype. Interestingly, in our large dataset we did not identify p.A382T, which is the most common variant in the Italian population and accounts for 30% of ALS, 0.9–2.5% of PD, and up to 6% of atypical parkinsonisms, as well as 0.5–1.3% healthy controls in Sardinia (Chiò et al., 2011; Quadri et al., 2011; Cannas et al., 2013).

The *TARDBP* mutations identified in our cohort were instead absent in 1,710 Italian healthy controls, including 771 individuals with no reported history of neurological disorders that we previously screened (Corrado et al., 2009) and 939 subjects enrolled in other case-control studies (del Bo et al., 2009; Origone et al., 2010; Conforti et al., 2011; Gagliardi et al., 2018). Moreover, all these variants resulted to be extremely rare in public sequencing databases and are classified as pathogenic or likely pathogenic according to the ACMG Criteria, with functional data in support of their pathogenicity (Table 1).

The pathomechanisms associated to these specific *TARDBP* mutations are not fully understood, although functional studies and *in silico* predictions strongly support their pathogenicity (Nonaka et al., 2009; Dewey et al., 2011; Sun et al., 2014; Kreiter et al., 2018; Table 2). Almost all the mutations identified in our study lead to the creation/disruption of serine residues, with potential consequences on TDP-43 aggregation and function. As TDP-43 is involved in multiple steps of RNA processing, a dysregulation of this protein may impact on many RNA and protein targets. Interestingly, a link between TDP-43 and microtubule-associated protein tau and alfa-synuclein, the major components of the intraneuronal deposits in parkinsonism, has been proposed, although more studies are needed to investigate if TDP−43 and these proteins may mutually promote the reciprocal accumulation. There is evidence that TDP-43 may act as regulator of tau exon 10 splicing, which generates the two isoforms 3R-tau and 4R-tau, in different cell types and in transgenic mice (Gu et al., 2017a). A dysregulation of exon 10 inclusion, possibly induced by mutated TDP-43 protein, may therefore lead to an altered ratio of 3R-tau/4R-tau, similarly to the effect induced by pathological *MAPT* mutations in exon 10. Additionally, TDP-43 appears to bind the 3′-UTR of *MAPT* promoting mRNA instability (Gu et al., 2017b). Moreover, in double transgenic mice, the overexpression of wild-type TDP-43 potentiates mutant α-synuclein toxicity leading in turn to a significant loss of dopaminergic neurons compared to both single transgenic mice (Tian et al., 2011). Unfortunately, no brain autopsy material was available for any of the mutated patients in order to evaluate the neuropathological profile and to test the extent and type of TDP-43 pathology.

Despite the relatively small sample size of the atypical parkinsonism group, our findings suggest that *TARDBP* mutations may also be associated to PSP and CBS, being observed in 1.4% of cases. A previous study on 67 Sardinian patients identified the founder *TARDBP* variant p.A382T in four patients with atypical parkinsonisms (Cannas et al., 2013), while another report described the p.N267S mutation in. a single CBS case (Huey et al., 2012; Supplementary Table 3). Therefore, given the relatively small number of atypical parkinsonism cases screened so far, it will be interesting to study larger cohorts to determine the role of *TARDBP* mutations in the pathogenesis off CBS and PSP.

## Conclusion

In conclusion, our study indicates that the clinical phenotypic presentation associated to *TARDBP* mutations, and in particular to the p.N267S variant, may expand beyond the classical ALS and FTD spectrum to include also PD and atypical parkinsonisms.

## Data availability statement

The datasets presented in this study can be found in online repositories. The names of the repository/repositories and accession number(s) can be found below: Zenodo (doi: 10.5281/zenodo.6985371).

## Ethics statement

The studies involving human participants were reviewed and approved by the IRCCS Istituto Auxologico Italiano. The patients/participants provided their written informed consent to participate in this study.

## Author contributions

CT, SG, GP, VS, and NT: study design. SG, AZ, and GP: data collection. CT, DC, FV, SP, DS, AR, and NT: genetic analysis. CT, SG, and NT: data analysis. CT, NC, SP, and NT: writing of first draft. SG and NT: supervision. All authors revised the manuscript for intellectual content.

## Footnotes

1
http://biobanknetwork.telethon.it/
