# Supplementary material for: TARDBP mutations in a cohort of Italian patients with Parkinson’s disease and atypical parkinsonisms
Source: Front Aging Neurosci. 2022 Sep 29;14:1020948. doi: 10.3389/fnagi.2022.1020948 (PMC9557978; doi:10.3389/fnagi.2022.1020948)
Supplement: Supplementary file 1 [file Data_Sheet_1.docx]

**SUPPLEMENTARY MATERIAL**

**Supplementary Table 1. Demographic and clinical features of Italian study population.**

| **Characteristic** | **Parkinson’s disease** | **Atypical parkinsonism** |
| --- | --- | --- |
| Patients, n | 735 | 142 |
| Male/Female (ratio) | 443/292 (1.5) | 77/65 (1.2) |
| Age at onset, years (mean ± SD) | 57.82±10.20 | 65.12±7.07 |
| Disease duration, years (mean ± SD) | 10.96±5.90 | 7.26±3.41 |
| Family history, n (%) | 354 (48.2%) | 12 (8.2%) |

n, number of cases, SD, standard deviation

**Supplementary Figure 1.** Sanger sequencing chromatograms of *TARDBP* exon 6 showing heterozygous missense variants in affected patients. Vertical black arrows indicate the mutated nucleotide.


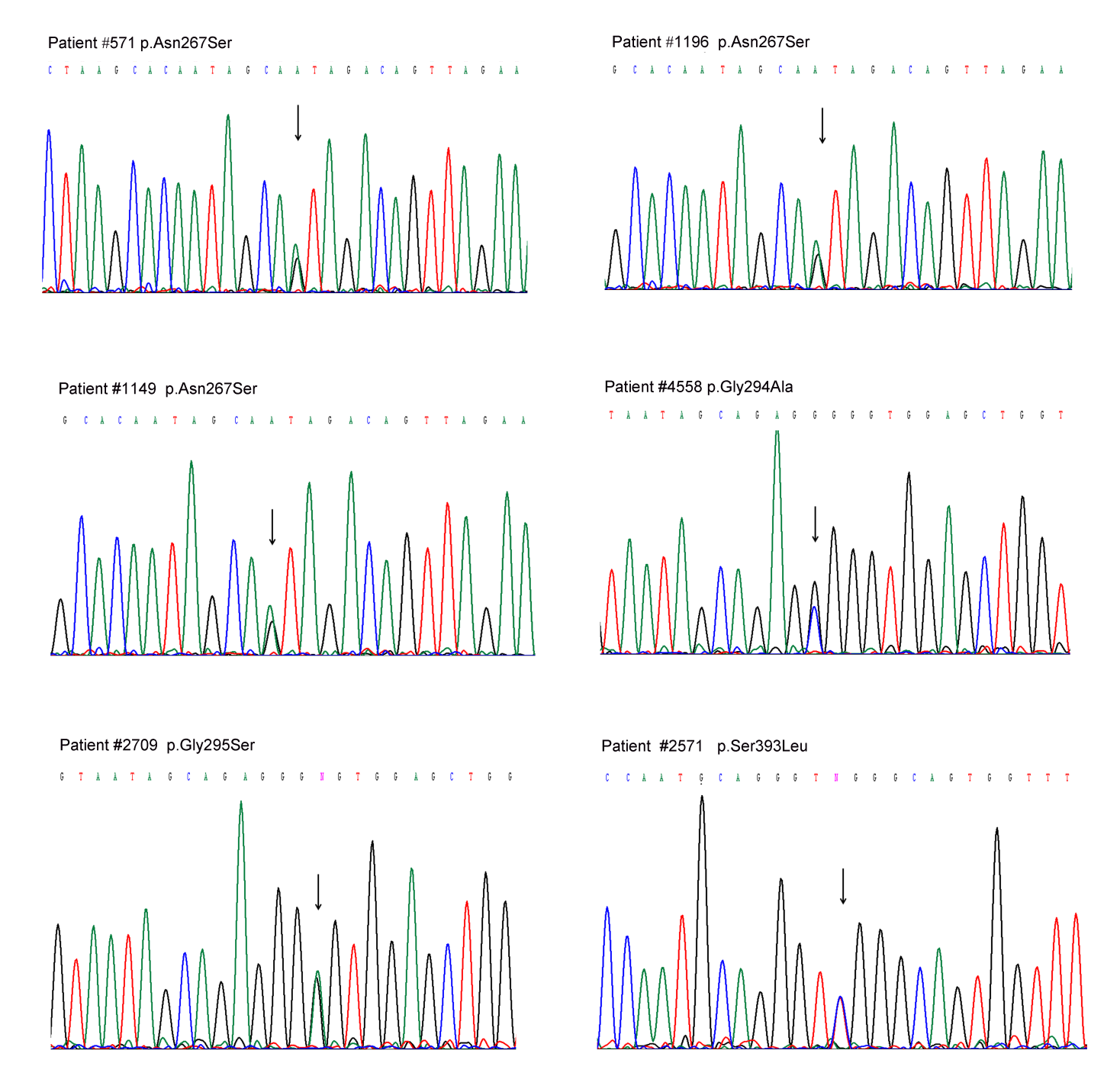


**Supplementary Table 2.** In silico analysis of *TARDBP* intronic and synonymous variants identified in our PD cohort.

| **Variant** | **SSF** | **MaxEnt** | **NNSPLICE** | **GeneSplicer** | **Human splicing finder** | **ESE Finder** |
| --- | --- | --- | --- | --- | --- | --- |
| c.715-31C>T | Acceptor site  = 86.72 | Acceptor site  = 7.78 | Acceptor site = 0.99 | 7.68 ⇒ 7.47 (-2.8%) | No difference between WT and mutant | No difference between WT and mutant |
| p.Gly335Gly |  |  |  |  |  | SRSF2 2.388⇒2.675 |
| p.Ala341Ala |  |  |  |  |  | SRSF1 2.05 ⇒ — |

Numbering of TARDBP variants according to the NCBI Reference Sequence NM_007375.3

For the variant c.715-31C>T values are reported according to canonical acceptor splicing site

Abbreviations: WT, wild-type; — absent or below threshold

**Supplementary Table 3.** Literature review of *TARDBP* mutations identified in PD patients and associated clinical features.

|  | | **TARDBP mutations** | | | | |
| --- | --- | --- | --- | --- | --- | --- |
|  |  | **p.N267S** | **p.G294A** | **p.G295S** | **p.A382T** | **p.S393L** |
| N. of PD patients | | 3 | 1 | 1 | 13 | 1 |
| Sex | *Female* | 0 | 1 | 1 | 3 | 0 |
|  | *Male* | 3 | 0 | 0 | 10 | 1 |
| Family history | *Familial PD* | 3 | 0 | 0 | 5 | 0 |
|  | *Sporadic PD* | 0 | 1 | 1 | 8 | 1 |
| Age at onset, years (mean ± SD) | | 51,0±12,7 | 58 | 47 | 68,3±9,8 | 67 |
| Disease duration, years (mean ± SD) | | n/a | 11 | 7 | n/a | 15 |
| Parkinsonian features | | RT (n=3, 100%) | RT | RT | RT (n=8, 62%) | RT |
|  |  | B (n=3,100%) | B | B | B (n=12, 92%) | B |
|  |  | R (n=3, 100%,) | R | R | R (n=11, 85%) | R |
|  |  | PI (n=1, 33%) | PI |  |  |  |

Numbering of TARDBP variants according to the NCBI Reference Sequence NM_007375.3 Abbreviations: B, bradykinesia; n/a, not available for all patients; PD, Parkinson’s disease; PI, postural instability; R, rigidity; RT, resting tremor.
